# The Library Part of the Journal

**Published:** 1849-07

**Authors:** 


*The Library Part of the Journal.*—We had hoped to have completed the publication of the translation of Jourdain's work in the present number of the Journal, but it will probably make one hundred pages in the next or tenth volume. The translator, Dr. P. H. Austen, has performed his task thus far, in a highly creditable manner, and for presenting to his professional brethren in this country, for the first time, this excellent and highly interesting work, in our own noble language, he has entitled himself to their lasting gratitude. He has very judiciously omitted, as will readily be perceived by those familiar with the work in its original language, such parts as possess no value at the present time, and supplied other parts with valuable additions and notes. The English, therefore, will be by far more valuable than the French edition.—*Balt. Ed.*

